# Effect of Physicochemical Characteristics and Storage Atmosphere on Microbiological Stability and Shelf-Life of Minimally Processed European Sea Bass (*Dicentrarchus labrax*) Fillets

**DOI:** 10.3390/foods12061145

**Published:** 2023-03-08

**Authors:** Dimitrios A. Anagnostopoulos, Foteini F. Parlapani, Evangelia Tsara, Maria G. Eirinaki, Despoina Kokioumi, Evdoxia Ampatzidou, Ioannis S. Boziaris

**Affiliations:** 1 Laboratory of Marketing and Technology of Aquatic Products and Foods, Department of Ichthyology and Aquatic Environment, School of Agricultural Sciences, University of Thessaly, Fytokou Street, 38446 Volos, Greece; 2 Kefalonia Fisheries, Livadi, Lixouri, 28200 Kefalonia, Greece

**Keywords:** sea bass, brining, marinating, minimal process, shelf-life, MAP, vacuum, microbiological spoilage, microbiological safety

## Abstract

The aim of the present work was to evaluate the effect of various hurdles such as a_w_ and pH as well as the storage atmosphere on the microbiological and sensory changes of minimally processed (lightly brined or marinated with acetic or citric acid) European sea bass (*Dicentrarchus labrax*) fillets. The results indicated that the shelf-life of brined fillets stored aerobically was 6 d while that of vacuum and MAP-stored was 12 d, since a reduced growth rate of spoilage bacteria was recorded. The physicochemical characteristics such as a_w_ and water phase salt (WPS) were not considerably changed, while the oxygen levels into the packages ensure the microbiological safety of the product. The fillets marinated with acetic acid exhibited a longer shelf-life at 30 and 40 d under aerobic and reduced oxygen conditions, respectively, while the products marinated with citric acid were at 25 and 35 d respectively. A low pH resulted in reduced or even limited microbial levels, especially for the fillets marinated with acetic acid; something that ensures microbiological safety as well. The low or limited microbial levels in conjugation with the sensory attributes indicated that spoilage may be due to other mechanisms such as autolysis rather than microbial activity. Overall, the present work highlights the potential for further research and development of minimally processed, microbiologically safe and stable with extended shelf-life value added seafood to satisfy the corresponding consumer demands.

## 1. Introduction

Seafood products constitute an important part of the human diet, mainly due to their high nutritional value and quality. Many seafood products are closely related to high-quality nutrition and human health promotion, since they are rich in proteins, vitamins, minerals and omega-3 polyunsaturated fatty acids [1,2]. Over the past two decades, the modern dietary trends that resulted in a remarkable growing demand for fish and fish products at a global scale has forced the aquaculture sector, which is now the main fish provider to the international commerce [3], to rapidly increase production in order to meet market needs. European sea bass (*Dicentrarchus labrax*) is among the most popular farmed fish worldwide, being a highly valued and appreciated seafood product, as well as a vital part of the Mediterranean diet [4]. 

Fish is a very perishable food, mainly due to an intense microbial activity and more specifically the metabolism of the so-called Specific Spoilage Organisms (SSOs) [5,6]. The study of fish spoilage, the monitoring and the determination of its shelf-life during various commercialized storage conditions and the development of targeted strategies to control the spoilage course are of great interest to extend its shelf-life and thus, ensure its quality and increase its added value [7]. Apart from the effort to achieve a longer shelf-life, various treatments based on the hurdle technology concept [8,9,10] lead to the creation of different kinds of products which offer a variety of added-value seafood products. 

Several interventions are used for fish preservation, including a reduced pH and water activity (a_w_). Lowering a_w_ is one of the most widely strategies applied to preserve fish products. The reduction of a_w_ by salting/brining inhibits bacterial growth and can also reduce the activity of various autolytic enzymes [11,12]. Salting is achieved by placing fish in salt (dry salting) or salt solution (brine). However, a potential use of high salt content is inconsistent with the recommendations of the World Health Organization [13] and consumer demands, since high levels of sodium chloride intake is associated with significant health issues and concerns. Thus, the processing of fish fillets only to low levels of exposure to sodium chloride make this process partially ineffective, at the expense of trying to increase the shelf-life of seafood products.

Lowering the pH by marination is another way of inhibiting microbial growth. Marinades are solutions of salt, lemon or vinegar [14]. Marinades can also include sugars, spices, oil and flavorings, in which fish fillets are immersed and used not only to retard bacterial growth and enzymic activity [11,12] but also to tenderize and improve the taste, textural and structural properties of raw fish [15]. However, marinated products must be stored under refrigeration conditions, to ensure the safety and microbiological stability of the products [16]. 

Extending the shelf-life of such lightly preserved products could possibly be achieved with the implementation of additional strategies. The use of a modified atmosphere enriched in carbon dioxide and reduced in oxygen or vacuum packaging as an extra hurdle is an option. It has to be pointed out that in reduced oxygen packaging, major safety issues related to anoxic conditions must be considered, since in such products there is the risk of *Clostridium botulinum* type E [17]. To tackle this, there are some options regarding the control of the product’s intrinsic properties such as a_w_, salt content and pH and/or the use of a high oxygen permeability package [18,19]. 

The aim of the present work was to study the microbiological stability, spoilage and shelf-life of lightly processed (brined or marinated with acetic or citric acid) European sea bass (*Dicentrarchus labrax*) fillets during storage at 4 °C at various atmospheric conditions. Such information will be very useful for food processors and other stakeholders to produce safe, added value products based on European sea bass of a high quality, as well as an extended shelf-life. 

## 2. Materials and Methods

### 2.1. Provision and Processing

European sea bass (*Dicentrarchus labrax*) of approximately 450 g were provided by Kefalonia Fisheries SA (Kefalonia, Greece). The fish were transferred to the processing unit immediately (at the same day), where they were filleted. The fillets (approximately 100 g each) were packed in insulated boxes with melted ice and transferred to the Laboratory of Marketing and Technology of Aquatic Products and Foods (University of Thessaly, Volos, Greece) within 24 h.

The fillets were divided into three groups. The first group was lightly brined fillets, and prepared by immersion in a brine solution containing 6% *w*/*v* NaCl for 3 h at 8 °C, with a fillet to brine ratio of 1:1.5 *w*/*v* (100 g of product was immersed in 150 mL of brine), with the intention to produce a ready-to-cook product. After that, the fillets were allowed to drain and dry for 1 h at 4 °C and divided into three packaging groups of about 30 samples each: (i) under aerobic conditions, (ii) in vacuum packaging and (iii) in modified atmosphere packaging (MAP) with a gas mixture containing CO_2_ (50%), O_2_ (10%) and N_2_ (40%). Plastic pouches (Intrama Protek Ltd., 9300 Dobrich, Bulgaria) were used with medium oxygen permeability (OTR 39.2 cm^3^/m^2^, 24 h, atm at 23 °C, and 0% relative humidity) to allow oxygen concentrations inside the packages to prevent the risk of *Clostridium botulinum* type E.

The second and third groups of fillets after skin removal were treated with the intention to produce a ready-to-eat product as following: One group of fillets was cooked in a pre-heated oven at 180 °C for 20 min. After that, the fillets were marinated in 3% *w/v* solution of commercial acetic acid (vinegar) with the addition of 3% *w/v* NaCl, and a ratio of product to marinade 1:1.5 *w/v* for 20 h at 8 °C. The other group of fillets was marinated in 3% *w/v* solution of commercial citric acid with the addition of 3% *w/v* NaCl, and a ratio of product to marinade 1:1.5 *w/v* for 20 h at 8 °C. After marination, the fillets of both groups were allowed to drain and dry for 1 h at 4 °C and divided into two packaging groups of about 40 samples each: (i) under aerobic conditions and (ii) vacuum packaging with the same plastic pouches of medium oxygen permeability film (OTR 39.2 cm^3^/m^2^, 24 h, atm at 23 °C, and 0% relative humidity).

After packaging, all samples were stored at 4 °C until the end of each product shelf-life. At the sampling points, the fillets were randomly selected for sensory, microbiological and physicochemical analyses.

### 2.2. Physicochemical Analysis

#### 2.2.1. Determination of pH and Water Activity (a_w_)

The pH values were taken using a pH meter (inoLab^®^ pH 730). The pH value of the products was measured after dilution and homogenization of 5 g of product with 50 mL distilled water. The determination of water activity (a_w_) was carried out using the HygroPalm HP23-A/HP23-AW-A Hand-Held Indicator machine (Rotronic, Switzerland).

#### 2.2.2. Determination of Water Phase Salt (WPS)

The WPS of the samples was calculated by taking into account the mean values of % salt and % moisture using the formula: WPS (%) = (% Salt × 100)/(% moisture +% Salt).

The determination of the salt content of fillets (% Salt) was performed following the Volhard method according to the AOAC for fishery products [20], by adding 30 mL AgNO_3_ (0.1 M) (PanReac AppliChem, Barcelona, Spain) and 20 mL HNO_3_ (0.1 M) (Ing. Petr Švec–PENTA s.r.o., Prague, Czech Republic) to 5 g of sample, followed by gentle heating (~15 min), until the solid residues dissolved and a total of 30 mL of distilled water and 3 mL of ferric indicator were then added. Finally, a titration with NH_4_SCN (0.1 M) (PanReac AppliChem, Barcelona, Spain) was carried out until the solution was steadily colored light brown.

Determination of moisture (% moisture) was also carried out after drying at 105 °C for 24 h. The results were expressed as mean ± standard deviation of three replicates.

#### 2.2.3. Determination of Gas Concentrations within the Packages

Gas concentrations (CO_2_ and O_2_) within the packages were also being monitored using a Dansensor^®^ CheckPoint^®^ 3 m (AMETEK MOCON, Minneapolis, MI, USA).

### 2.3. Sensory Evaluation

Sensory evaluation of the brined, ready-to-cook product was applied after cooking for 20 min in a preheated at 180 °C oven and, before being tested, they were allowed to cool down to the appropriate temperature of a cooked product. The other two products were ready to eat and were tested without cooking, after allow them to cool at room temperature for 15 min. The appearance, texture, odor and taste were evaluated by a panel of five (5) trained persons who scored using a descriptive hedonic scale from 5 to 1, with 5 to be excellent, 4 good, 3 accepted, 2 not accepted, while 1 was the spoiled sample. The product was rejected when one out of the five panelists scored one of the sensory attributes with two (2).

### 2.4. Microbiological Analysis

A microbiological analysis was also carried out. Briefly, twenty-five grams (25 g) of product were transferred aseptically to stomacher bags with 225 mL MRD (Maximum Recovery Diluent, 0.1% *w/v* peptone, 0.85% *w/v* NaCl) and homogenized for 2 min using a Stomacher (Bug Mixer, Interscience, London, UK). Using the spread plate technique, an amount of 0.1 mL of 10-fold serial dilutions was applied on the surface of dried media in Petri dishes for the enumeration of Total Viable Counts (TVC) on TSA (Tryptone Soy Agar), incubated at 25 °C for 48–72 h, and *Pseudomonas* spp. on a cetrimide–Fucidin–cephaloridine agar (CFC) incubated at 25 °C for 48 h. Using the pour plate technique, 1 mL of the 10-fold serial dilutions was used for the enumeration of H_2_S producing bacteria (presumptive *Shewanella* spp.) on iron agar lyngby (IA) by counting only the black colonies after incubation at 25 °C for 72 h, Enterobacteriaceae on violet red bile glucose agar (VRBGA) after incubation at 37 °C for 24 h and lactic acid bacteria (LAB) on De Man, Rogosa, Sharpe agar (MRS), incubated at 30 °C for 72 h. All microbiological media were supplied from LAB M (Lancashire, UK) while IA was made by its ingredients according to [21]. The results were expressed as mean log cfu g^−1^ ± standard deviation (log colony forming unit per g) of three replicates per treatment.

### 2.5. Statistical Analysis

The differences of means were subjected to an Analysis of Variance (one-way ANOVA) followed by Tukey’s post hoc test using the IBM^®^ SPSS^®^ statistics 19 software (SPSS Inc., Chicago, IL, USA) and a probability level of *p* ≤ 0.05 was considered statistically significant.

## 3. Results

### 3.1. Physicochemical Characteristics

**Figure 1:**
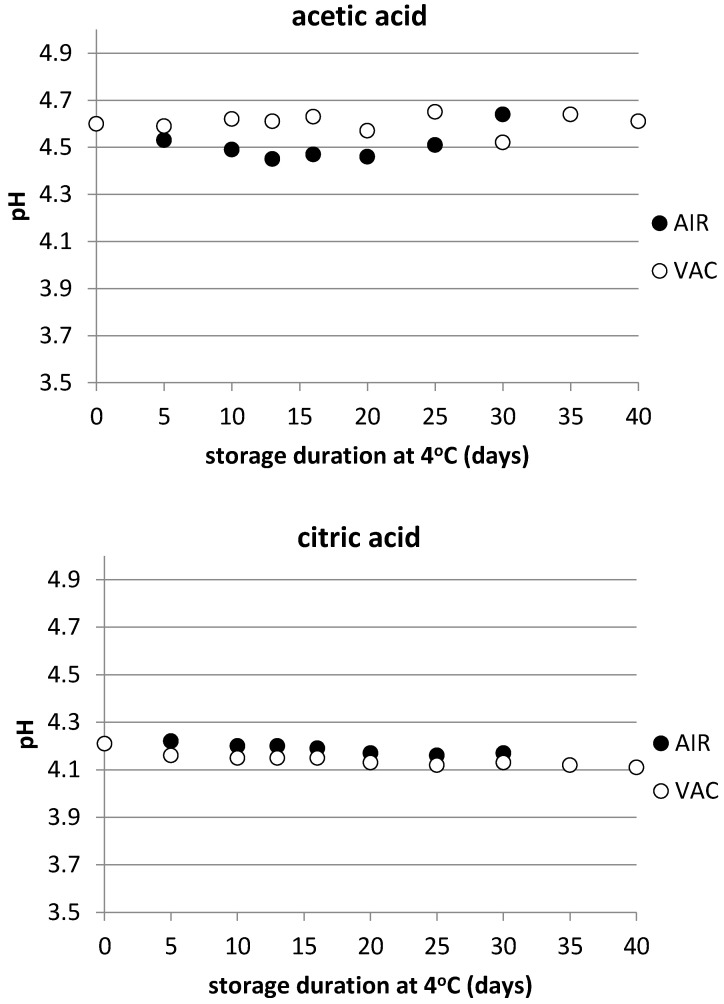
Changes of pH in sea bass fillets marinated with acetic and citric acid stored under air (AIR) and vacuum (VAC) conditions.

The physicochemical parameters of the finished products are shown in Table 1. The physicochemical parameters were also monitored throughout storage; more specifically, the water activity (a_w_) and WPS for the brined product and the pH for the marinated ones, which are the important parameters for their microbiological stability (Table 2 and Figure 1).

Initially (day 0), the physicochemical attributes of the brined product were characterized by a pH value of 6.2, a water activity close to 0.972, while WPS was 2.85% (Table 1). On the other hand, both marinated products had a pH value below 5.0. More specifically, for the fillets marinated in acetic and citric acid, the pH values were 4.6 and 4.2, respectively. The a_w_ was about 0.98 for both products, quite high compared to the brined one, something that was expected since the WPS was 1.20 and 1.13% for the fillets marinated in acetic and citric acid, respectively (Table 1).

In the following days, the brined fillets exhibited similar water activities, which were not lower than 0.97 throughout storage (Table 2). Accordingly, the calculated WPS varied in an arbitrary way (from a minimum of 2.63 to a maximum of 3.33%), which can be attributed to the expected experimental deviations (Table 2). Indeed, the differences in % moisture and % salt were not significantly different (*p* > 0.05).

The pH values were also monitored throughout storage for the marinated products and did not exhibit important differences to be considered from the point of view of microbiological stability. Indeed, the pH value of the product marinated in acetic acid was found to fluctuate between 4.4 and 4.7 (initial value of 4.6-Table 1) throughout storage in both the aerobic and vacuum conditions (Figure 1). The pH value of the product marinated in citric acid was more stable, since it did not fluctuate but slightly dropped to 4.1 from the initial value of 4.2 (Table 1), in both the aerobic and vacuum conditions (Figure 1).

### 3.2. Gas Changes throughout Storage

The concentrations of the gas mixture in the MAP, as well as in the vacuum packaging, are presented in Table 3. Throughout the storage period, oxygen was present to some extent, even in vacuum packaging. More specifically, under the MAP conditions of the brined product, the concentration of CO_2_ was reduced as the storage time passes from an initial value of about 50.1% (day 0) to 4.2% at the end of the fillets shelf-life (at 12th day), while oxygen was increased from 9.9% to 18.6% at the end of the brined fillets shelf-life. Furthermore, in the vacuum-stored samples, the oxygen concentration was increased from 0.1% to 13.7% at the end of the brined product shelf-life, while at the late stages of the storage of marinated products, oxygen reached a level the same as the atmospheric air (Table 3).

### 3.3. Sensory Analysis and Determination of Shelf-Life

Initially, the sensory attributes of the fillets were excellent (Grade 5), with an appropriate appearance, good texture and excellent odor and very desirable taste. More specifically, the brined ones had a slight and desirable salty taste, while the marinated had the distinct taste of marinated fish in acetic or citric acid. However, these characteristics were degraded with the passage of storage time and at the rejection time point the texture and some unpleasant odors and/or taste were the most obvious characteristics indicating spoilage. More specifically, for the brined cooked samples, unpleasant fishy odors and an undesirable slightly putrid taste were the most obvious characteristics indicating the rejection. For the cooked fillets marinated in acetic acid, the rejection occurred mostly due to the softness during chewing and the development of a slight bitter taste, while the appearance and odor was still acceptable. Similarly, the fillets marinated in citric acid developed a softness and undesirable odor and taste, while the appearance was still acceptable.

The products reached the rejection level at different time points. More specifically, for brined fillets the rejection occurred at day 6 for the air-stored fillets and at day 12 for both the vacuum and MAP-stored ones. On the other hand, the end of the shelf-life of the cooked fillets marinated with acetic acid was determined at 30 and 40 days for air and vacuum-stored products, respectively, while the fillets marinated with citric acid were rejected at 25 and 35 days stored under aerobic and vacuum conditions, respectively (Table 4).

### 3.4. Microbiological Analysis

**Figure 2:**
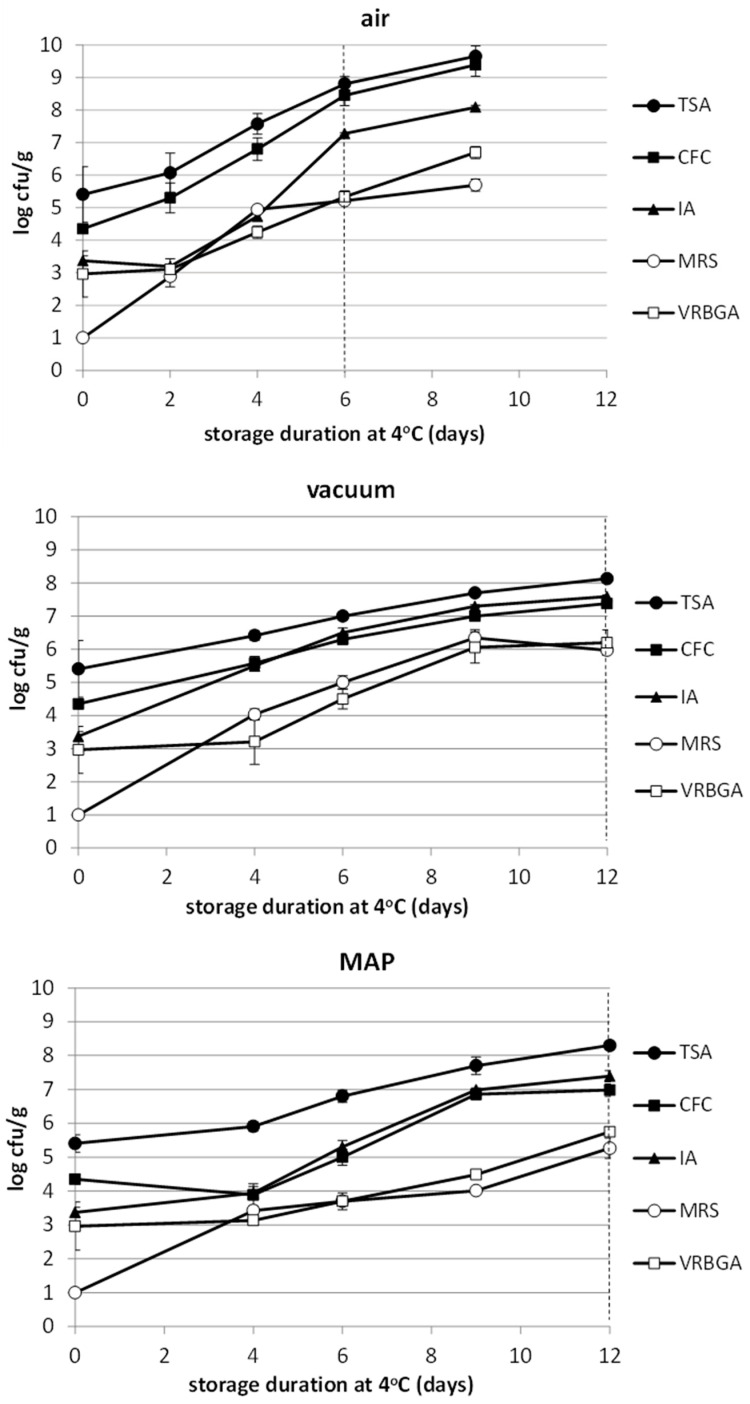
Changes of microbial populations in brined sea bass fillets stored under air vacuum and modified atmosphere (MAP) conditions. Each point is the mean of three replicates ± standard deviation. The dashed vertical line indicates the sensory rejection time point.

Regarding the microbiological profile of the brined fillets, the results indicated that the initial levels of total viable counts (TVC) on TSA (day 0) was about to 5.5 log cfu/g (Figure 2). The TVC levels increased rapidly, but at a faster rate in the fillets stored under aerobic conditions reaching, at the end of shelf-life (day 6), population levels close to 8.8 log cfu/g. On the other hand, the TVC in the fillets stored under both the vacuum and MAP conditions increased at a slower rate, reaching levels at the end of the shelf-life (day 12) of about 8.1 and 8.4 and log cfu/g, respectively (Figure 2). *Pseudomonas* spp. were the dominant microorganisms with an initial population (day 0) close to 4.5 log cfu/g, and increased rapidly, especially in the aerobic condition (Figure 2). At the rejection time point, the air-stored fillets (day 6) showed a *Pseudomonas* spp. population level close to 8.5 cfu/g, while MAP-stored and vacuum-stored fillets (day 12) reached levels of about 7 and 7.3 log cfu/g, respectively. The presence of H_2_S-producing bacteria was also remarkable in all samples, reaching populations of above 7 log cfu/g at the end of each product shelf-life in all cases, with no significant differences between them (Figure 2). The presence of lactic acid bacteria (LAB) was also notable, recording levels of more than 5 log cfu/g at each product’s end of shelf-life. It is crucial to mention that, meanwhile (day 9), the MAP-stored fillets exhibited significantly lower populations compared to both the air and vacuum-stored ones. The same tendency was also observed for the bacteria belonging to the family of Enterobacteriaceae, even though no significant differences were recorded between the products at the end of their shelf-life. It should be mentioned that the microbial population rates in both MAP- and vacuum-stored samples were lower than that of the air-stored ones (Figure 2).

**Figure 3:**
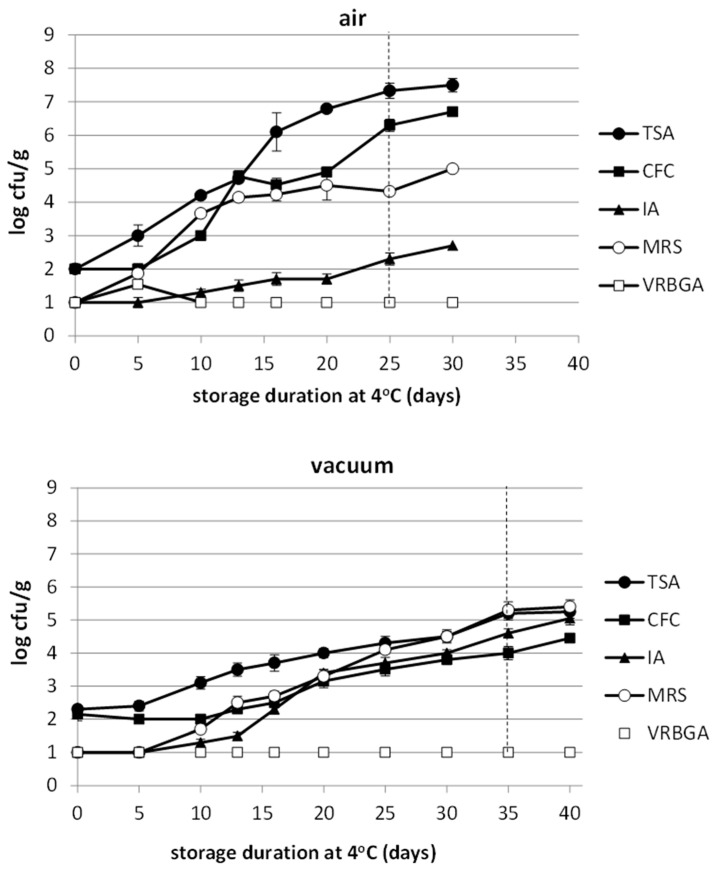
Changes of microbial population in sea bass fillets marinated with citric acid, stored under air and vacuum conditions. Each point is the mean of three replicates ± standard deviation. The dashed vertical line indicates the sensory rejection time point.

The use of citric acid not only eliminated the initial microbial population, but also inhibited the microbial growth. More specifically, the initial level of TVC (day 0) was close to the detection limit of 2 log cfu/g (Figure 3). During storage, the TVC was increased at a slow rate but this rate was faster in aerobically stored marinated fillets. At the end of shelf-life (day 25 and 35 for the air-stored and vacuum-stored products, respectively), the population level was close to 7.3 and 5.2 log cfu/g for air-stored and vacuum-stored fillets, respectively. Similarly, *Pseudomonas* spp. growth was more remarkable at the late stages of the storage of air-stored fillets, reaching levels of about 6.3 log cfu/g at the end of shelf-life (day 25), while their growth was limited in vacuum-stored fillets reaching a level of 4.0 log cfu/g at the end of shelf-life (day 35) (Figure 3). On the other hand, the presence of H_2_S-producing bacteria was remarkable only in the vacuum-stored samples, reaching levels of more than 4.5 log cfu/g at the end product shelf-life (day 35), while the population in the air-stored fillets was really low throughout storage (Figure 3). LAB were present in both packaging conditions (Figure 3). Despite that, their growth rate was more limited in vacuum-stored samples; they were the dominants at the end of shelf-life with a population level of about 5.2 log cfu/g. Finally, the Enterobacteriaceae population remained below the detection limit of 1 log cfu/g in both packages, throughout storage.

The microbial presence and growth in the cooked and then marinated in acetic acid fillets was almost negligible throughout storage, under both aerobic and anaerobic conditions. The population levels in the vacuum-stored products were below the detection limits throughout storage (day 40). All bacterial populations of the air-stored fillets were below the detection limit for most of the storage time and were only seen at the last days of storage growth with TVC, *Pseudomonas* spp. and H_2_S-producing bacteria to reach levels of about 5, 4 and 3 log cfu/g, respectively, at the end of shelf-life (day 30).

## 4. Discussion

The physicochemical characteristics of minimal processed foods stored at chilling temperatures, especially under reduced oxygen conditions, are essential to ensuring microbiological stability/safety and extending the shelf-life.

Regarding the lightly salted, ready-to-cook product, the shelf-life at 4 °C under aerobic conditions was 6 days, while in reduced oxygen packages, it was 12 days. Under aerobic conditions, the inhibition of spoilage bacteria due to an elevated salt content and reduced a_w_ was negligible compared to fresh fish, where a shelf-life at 4 °C under aerobic conditions with similar initial microbial populations did not differ considerably [22,23,24,25,26,27]. Furthermore, *Pseudomonas* spp. was the dominant spoilage microorganism as it is in fresh chilled stored fish [24,26,27,28,29,30,31]. The combination of storage with reduced oxygen doubled the shelf-life of the product since both the growth rates and maximum population densities were lower. Reduced oxygen in the packages also affected the microbial spoilage association, with *Shewanella* spp. to be co-dominant with *Pseudomonas* spp., while lactic acid bacteria were favored in vacuum packages, something that is expected and has been seen for various types of fresh or lightly preserved seafood in such packaging conditions [32,33,34,35,36,37].

It must be noted that In the lightly brined product, the a_w_ and WPS values were above and below, respectively, from those required to prevent the risk of *Clostridium botulinum* Type E in reduced oxygen packages with a shelf-life more than 10 days [17,18] FDA 2021). Indeed, the pH of the brined product obviously was not below 5, while the a_w_ was just above 0.97 and WPS quite below 3.5%. However, both the MAP and vacuum packages allowed oxygen presence in considerable amounts throughout storage, especially for the MAP where the oxygen was present at quite high amounts from the beginning of storage; hence, the microbiological safety of the product especially under MAP is ensured for a storage duration above 10 days. Alternatively, increasing the salt in the product would allow packages with less or even no oxygen, something that might considerably increase the shelf-life, but high amounts of salt content is not harmonized with the global guidance for human health, being closely linked to major health issues, such as hypertension and cardiovascular diseases [13]. On the other hand, the aim of this work was to study the development of a lightly salted product for sensory reasons; a ready-to-cook and definitely safe, value-added European sea bass product.

The marinated products were far more stable with an extended shelf-life, something that was expected due to the low pH of the products. The marinated in citric acid product revealed microbial growth of various fish spoilage bacteria in contrast to the marinated in acetic acid one, where the microbial growth was limited and apparent only at the end of storage. It is known that acetic acid exhibits greater antimicrobial activity compared to citric acid [37,38], hence its effect against microbial growth and the extension of shelf-life is more pronounced. The microbial spoilage association of marinated with citric acid and packed under a vacuum was totally different, with LAB to be dominant in contrast to *Pseudomonas* spp. and *Shewanella* spp., which were the dominants in the aerobically stored marinated with citric acid and brined product. This is obvious due to the ability of LAB to tolerate both low acidity and reduced oxygen, compared to other food spoilage bacteria; thus, it has also been found to dominate in many marinated fish products [32,33,39].

It must be noted that the microbial spoilage level of marinated in citric acid stored under vacuum products was reduced (10^5.5^ cfu/g), compared to the aerobically stored ones (10^7^ cfu/g), to the lightly brined product (10^8.5^ cfu/g) or even to any aerobically stored fresh fish which varies from 10^7^ to 10^9^ cfu/g [6]. It must also be noted the absence of noteworthy microbial growth in the marinated with acetic acid product. This implies that the main spoilage mechanism that determines shelf-life in these products might be autolytic or other, but not microbial. This argument is enhanced by the fact that the sensory results showed that the attributes that determined the shelf-life in the marinated products were mostly the unpleasant texture (softness) and the undesirable taste and not the unacceptable odor which is mostly an attribute of microbial spoilage. Indeed, such inferences have been drawn in other studies as well, where various hurdles that inhibit or eliminate microbial growth allow other spoilage mechanisms to take over and determine the shelf-life [12,40,41]. 

The marinated products had a pH well below 5, making the risk of *Clostridium botulinum* Type E, negligible; for this reason, the MAP with oxygen inclusion was not used. The main risk of these products could be other food-borne pathogens with the most important the *Listeria monocytogenes*, but the low pH of our products can definitely inhibit growth and might be able to inactivate such bacteria [42,43,44].

## 5. Conclusions

Overall, the present work provides useful information for food processors and other stakeholders to produce safe, added value products based on European sea bass of high quality, as well as of an extended shelf-life. The application of the hurdle technology concept enables a wide range of variables (parameters such as pH, a_w_, CO_2,_ etc.) to be used; however, the selection of the appropriate variables and their levels is crucial for the formulation of a sensorially accepted, safe and with prolonged shelf-life product. This study indicates that the combination of minimally processing based on the application of hurdles such as a_w_ and pH can give new added value products based on European sea bass fillets, which can be microbiologically safe and stable with a prolonged shelf-life under chilling temperatures. Especially, the combination of low oxygen packaging leads to an extended shelf-life compared to aerobic storage. The main issue in this case is the potential risk of *Clostridium botulinum* Type E, which can be prevented by ensuring the presence of adequate oxygen into the packages and/or the appropriate intrinsic factors such as a_w_ and pH, or other preservatives. The involvement and the combination of additional preservation systems, especially the use of natural antimicrobials and/or bioprotective cultures with the ability to produce various metabolites and/or bacteriocins against food-borne pathogens and spoilage bacteria, may greatly contribute towards this attempt and undoubtedly deserves much more attention by the food industry.

## Figures and Tables

**Table 1:** Physicochemical parameters of the final products. The measured values expressed as mean of three replicates ± standard deviation.

Product	pH	a_w_	%Moisture	% Salt	Calculated WPS%
Brined sea bass fillets	6.20 ± 0.01	0.972 ± 0.002	75.54 ± 1.17	2.22 ± 0.36	2.85
Cooked and marinated with acetic acid	4.60 ± 0.03	0.981 ± 0.006	77.37 ± 0.77	0.94 ± 0.03	1.20
Marinated with citric acid	4.21 ± 0.02	0.980 ± 0.003	77.54 ± 1.17	0.89 ± 0.02	1.13

**Table 2:** Changes during storage of water activity (a_w_), moisture % (*w/w*), salt content (% *w/w*) and NaCl content in the aqueous phase (Water Phase Salt–WPS%), in brined sea bass fillets during storage under air, vacuum and MAP conditions. The measured values expressed as mean of three replicates ± standard deviation.

Day	a_w_	% Moisture	% Salt	Calculated WPS%
Air				
4	0.972 ± 0.002	76.60 ± 1.76	2.35 ± 1.69	2.97
6	0.974 ± 0.003	77.96 ± 2.00	2.11 ± 0.58	2.63
Vacuum				
4	0.973 ± 0.002	76.23 ± 2.32	2.53 ± 0.49	3.21
9	0.971 ± 0.004	77.37 ± 1.97	2.67 ± 1.39	3.33
12	0.972 ± 0.003	75.89 ± 1.93	2.44 ± 1.01	3.11
MAP				
4	0.972 ± 0.003	74.92 ± 1.66	2.49 ± 0.53	3.22
9	0.973 ± 0.002	77.80 ± 7.76	2.28 ± 0.42	2.85
12	0.973 ± 0.005	76.01 ± 0.19	2.13 ± 0.22	2.73

**Table 3:** Gas changes (%) during storage of products under MAP and vacuum conditions.

Storage Day	MAP (CO_2_/O_2_) ^1^	Vacuum (CO_2_/O_2_) ^1^	Storage Day	Vacuum (CO_2_/O_2_) ^2^
0	50.1/9.9	0.0/0.1	0	0.0/0.1
4	23.6/12.1	0.4/4.1	12	0.3/11.6
9	9.4/15.5	0.3/10.4	25	0.3/18.4
12	4.2/18.6	0.2/13.7	40	0.3/20.1

^1^ Packaging of brined fillets. ^2^ Packaging of marinated fillets.

**Table 4:** Rejection time points of the products, stored at 4 °C according to the sensory evaluation.

Sample	Shelf-Life (Days)	Shelf-Life Determinants
Brined sea bass fillets (Air)	6	Unpleasant
Brined sea bass fillets (Vacuum) Brined sea bass fillets (MAP)	12 12	odor and taste
Cooked sea bass fillets marinated with acetic acid (Air)	30	Softness and
Cooked Sea bass fillets marinated with acetic acid (Vacuum)	40	bitter taste
Sea bass fillets marinated with citric acid (Air)	25	Softness and unpleasant
Sea bass fillets marinated with citric acid (Vacuum)	35	odor and taste

## Data Availability

The datasets generated for this study are available on request to the corresponding author.
